# mNeonGreen aggregates when overexpressed in *C. elegans* neurons

**DOI:** 10.17912/micropub.biology.001124

**Published:** 2024-02-08

**Authors:** Maithili Joshi, Sanne J. A. van Falier, Tessa Sinnige

**Affiliations:** 1 Bijvoet Centre for Biomolecular Research, Utrecht University, Utrecht, Utrecht, Netherlands

## Abstract

The optical transparency of the nematode *Caenorhabditis elegans* makes it possible to monitor the behaviour of fluorescently labelled proteins in a living multicellular organism. This study investigates the suitability of mNeonGreen as a fluorescent tag for studying proteins of interest in the nervous system of adult *C. elegans* . Despite its reported brightness, stability, and monomeric nature, our findings reveal that mNeonGreen forms solid aggregates in *C. elegans* neurons, particularly upon plasmid overexpression. We anticipate that this property may lead to artefacts when analysing for example the subcellular distribution or turnover of a tagged protein of interest.

**Figure 1. mNeonGreen forms solid aggregates when overexpressed in C. elegans neurons:**
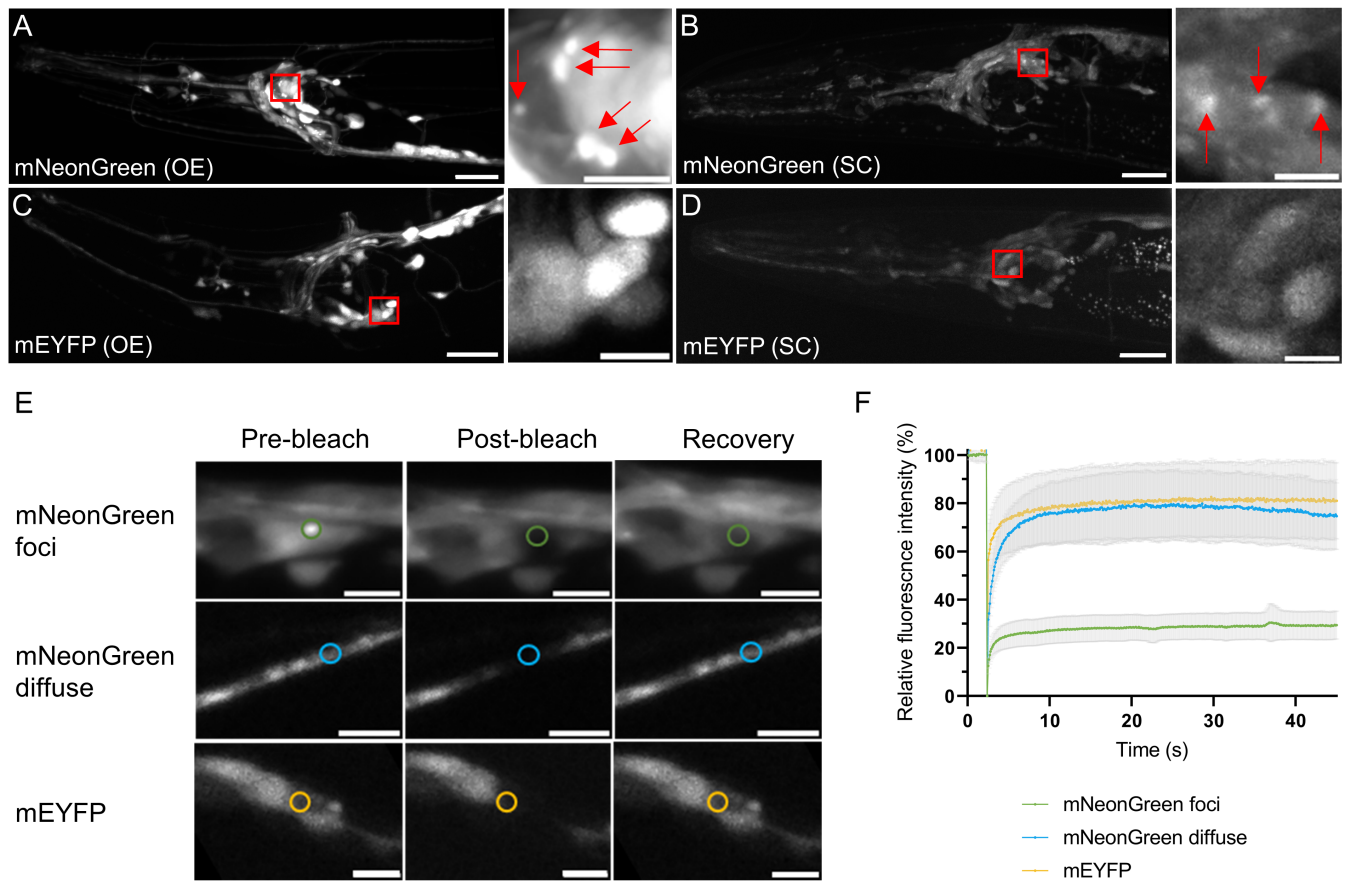
(A-D) Confocal images of *C. elegans* head regions expressing (A) mNeonGreen from plasmid overexpression (OE), (B) mNeonGreen from a single gene copy (SC), (C) mEYFP from plasmid overexpression, (D) mEYFP from a single gene copy. On the right, magnified images show mNeonGreen aggregates indicated by arrows. Scale bars: 20 μm (left panels), 5 μm (right panels). (E) FRAP of mNeonGreen foci (top), mNeonGreen diffuse signal (middle) and mEYFP (bottom). FRAP experiments were done on overexpression strains. The photobleached regions are marked by circles. Scale bars: 5 μm. (F) Quantification of fluorescence recovery. N = 9-14 animals per condition, data shown are average ± standard deviation.

## Description

Martin Chalfie’s success in expressing green fluorescent protein (GFP) in the nematode *Caenorhabditis elegans* (Chalfie et al., 1994) opened up new avenues to visualise the behaviour of proteins in a multicellular organism. *C. elegans* is optically transparent and the only multicellular model organism allowing for fluorescence microscopy in live animals throughout their lifespan. Fluorescent proteins can be fused to proteins of interest to study for example their expression pattern, subcellular localisation, trafficking, and interactions in live nematodes.

GFP, obtained from the jellyfish *Aequorea victoria* , has been widely used in *C. elegans* ever since the first demonstration. Its derivative yellow fluorescent protein (YFP) is another commonly used tag for studying proteins *in vivo* . YFP has the advantage that typical filter sets have a narrower bandwidth than for GFP, which reduces background signal and makes identification of transgenic animals easier. Both GFP and YFP are reported to have the tendency to dimerise (Yang et al., 1996) , yet this can be obliterated by a point mutation (Zacharias et al., 2002) . mNeonGreen is a more recently reported fluorescent protein derived from the lancelet *Branchiostoma lanceolatum* , whose monomeric nature was confirmed by size exclusion chromatography (Shaner et al., 2013) . One study showed the protein to be 3-5 times brighter than GFP when expressed in *C. elegans* (Hostettler et al., 2017) , while others contradict this observation (Heppert et al., 2016) .

Here, we unexpectedly encountered that mNeonGreen aggregates in *C. elegans* neurons *. C. elegans* strains were made expressing mNeonGreen throughout the nervous system either at overexpression or single-copy levels. Confocal imaging of the head region of adult transgenic *C. elegans* showed fluorescence in the neurons as expected ( Figure 1A, B ). However, small foci were apparent in some of the cell bodies ( Figure 1A ). The diameter of these foci was found to be 0.83 ± 0.26 μm (average ± standard deviation, N = 14). This feature was also observed at single-copy level, but not as pronounced ( Figure 1B ). Hence, the punctate appearance seems to be dependent on the concentration of mNeonGreen protein present. We compared these strains to those expressing monomeric enhanced YFP (mEYFP) ( Figure 1C, D ) and observed that even at overexpression levels, the mEYFP signal remains diffuse and intensity differences can be attributed to some cells being brighter than others ( Figure 1C ).

To further understand the nature of fluorescent foci formed by mNeonGreen, we carried out fluorescence recovery after photobleaching (FRAP) experiments in the overexpression strains ( Figure 1E, F ). The diffuse mNeonGreen signal recovers after bleaching, just as the mEYFP signal does ( Figure 1E ). However, when the foci are bleached, recovery is limited to ca. 30 % ( Figure 1F ). These results suggest that the foci formed by mNeonGreen are solid in nature and correspond to protein aggregates. We also considered the possibility that the foci may be lysosomes, in which certain fluorescent proteins have been shown to accumulate (Katayama et al., 2008) . However, we observed foci formation by the dimerisation-prone EYFP as well, although to a lesser extent than by mNeonGreen (Extended Data Figure S1). The fluorescence of EYFP is quenched at low pH, thus making it unlikely that the foci are lysosomes.

The behaviour of mNeonGreen should be taken into account when using it as a tag, since its aggregation can be expected to interfere with the localisation, function and biophysical properties of tagged proteins of interest. The aggregation propensity of mNeonGreen has not been reported thus far, and contradicts a previous *in vitro* study where the protein was shown to be monomeric (Shaner et al., 2013) . However, a similar contradiction has been reported for TagRFP which was found to be monomeric *in vitro* (Merzlyak et al., 2007) , whereas another study demonstrated strong oligomeric tendencies in cells (Costantini et al., 2012) . Also mCherry, which was derived from a monomeric red fluorescent protein (Campbell et al., 2002; Shaner et al., 2004) is notoriously aggregation prone *in vivo* (Melentijevic et al., 2017) .

It should be noted that the mNeonGreen foci must consist of folded protein, given that its fluorescence is preserved. Using fluorescence microscopy we cannot detect other types of aggregate species such as amyloids and amorphous precipitates, which have previously been reported for superfolder GFP (Stepanenko et al., 2021) . Altogether, we suggest caution when working with mNeonGreen in *C. elegans* neurons, and recommend using monomeric GFP derivatives instead.

## Methods

**Molecular biology**

Plasmids (Table 1) were generated using Gateway cloning and Gibson assembly (NEB). PCR products for the promotor region of *rgef-1* and the 3’-UTR sequence of *let-858* were amplified from *C. elegans* genomic DNA. The mNeonGreen fragment was amplified from plasmid pDD346 which was a gift from Daniel Dickinson (Addgene plasmid # 133311 ; http://n2t.net/addgene:133311 ; RRID:Addgene_133311). The EYFP fragment was amplified from pPD30.38 which was a gift from Andrew Fire (Addgene plasmid # 1443 ; http://n2t.net/addgene:1443 ; RRID:Addgene_1443). The A206K substitution was made using the Q5 site-directed mutagenesis kit (NEB). The amino acid sequences of mNeonGreen, EYFP and mEYFP correspond to those reported on FPbase (Lambert, 2019) . The inserts were assembled into the pDEST plasmid backbone using either Gateway cloning or Gibson Assembly according to the manufacturer’s protocol. All constructs were verified by Sanger sequencing.

***C. elegans* strain generation**

*C. elegans* overexpression strains were generated by injecting 30 ng/µL plasmids of interest (pMJ02 or pMJ19) supplemented with 70 ng/µL 1 kb DNA ladder (NEB) into the gonad of wild type ( N2 ) adults. Transgenes were integrated by treating fluorescent L4-staged animals with 30 mJ/cm ^2^ UV light using a UVP Crosslinker CL-3000 (Analytik Jena). UV-treated animals were incubated at 20 °C for 2 weeks and fluorescent offspring were singled and screened to obtain integrant lines. These were backcrossed with N2 worms five times to dilute UV-induced mutations. For single-copy strains, the MosSCI method was used (Frøkjær-Jensen et al., 2008). In brief, injection mix containing 10 ng/µL plasmid of interest (pMJ06 or pMJ20), 50 ng/µL pCFJ601 (transposase), 10 ng/µL pMA122 ( *peel-1* ) and 30 ng/µL ccGFP (co-injection maker) was injected into the gonads of EG6699 adults. These were incubated at 25 °C for one week followed by a 2 h heat shock at 34 °C. The plates were chunked and screened for fluorescent animals with rescued movement and lacking expression of the co-injection maker. The insertion of the transgene was confirmed by amplifying the insert using PCR with LongAmp Taq DNA polymerase (NEB) followed by sequencing. These animals were also backcrossed with N2 five times to dilute mutations induced by the heat shock.

***C. elegans* strains and maintenance**

All strains (Table 2) were maintained on nematode growth media (NGM) seeded with *Escherichia coli* OP50 at 15 °C. Age-synchronised populations for confocal microscopy were obtained by allowing adult animals to lay eggs for 1 h at 20 °C, followed by 3 days of incubation at 20 °C to reach day 1 of adulthood.

**Microscopy and FRAP**

For the selection of transgenic animals, Leica MZ10F and Leica M165FC stereomicroscopes were used with filter set ET YFP with excitation 500/20 nm and emission 535/30 nm. Samples for confocal imaging were prepared by immobilising day 1 adult animals on 2.5 % agarose pads in a drop of 10 mM tetramisole in M9 (22 mM KH _2_ PO _4_ , 42 mM Na _2_ HPO _4_ , 8.5 mM NaCl, 18.7 mM NH _4_ Cl, 1 mM MgSO _4_ ). Imaging was done on a Nikon Eclipse Ti microscope body equipped with CSU-X1-A1 spinning disk (Yokogawa) and Nikon Plan Apo VC 60x /1.40 oil objective. The excitation and the emission wavelengths used were 488 nm and 525/50 nm, respectively. The laser intensities used for single-copy and overexpression strains were 30 % and 5 %, and the exposure times 250 ms and 100 ms, respectively. For FRAP on overexpression strains, regions of interest were photobleached for 200 ms at 100 % laser intensity and the recovery was monitored for 45 s.

## Reagents

**Table 1.** Plasmids used in this study.

**Table:** 

Plasmid	Description
pDEST R4-R3 Vector II	Destination vector with ampicillin resistance for overexpression strains
pCFJ150- pDEST *ttTi5605* [R4-R3]	Destination vector with ampicillin resistance for single-copy strains
pMJ02	Pan-neuronal expression of mNeonGreen, back-bone: pDEST
pMJ06	Pan-neuronal expression of mNeonGreen, back-bone: pCFJ150
pMJ19	Pan-neuronal expression of mEYFP, back-bone: pDEST
pMJ20	Pan-neuronal expression of mEYFP, back-bone: pCFJ150
pMA122	Expression of *peel-1* toxin, negative selection maker
pCFJ601	Expression of transposase enzyme
ccGFP	GFP expression in coelomocytes, co-injection marker

**Table 2.** *C. elegans* strains used in this study.

**Table:** 

Strain	Genotype	Available from
N2	*C. elegans* wild isolate	CGC
EG6699	*ttTi5605 II; unc-119 ( ed3 ) III; oxEx1578 [eft-3p::GFP + Cbr- unc-119 (+)] *	CGC
TSW26 (mNeonGreen overexpression)	*mbbIs4 [ rgef-1 p::mNeonGreen:: let-858 -3’UTR] *	This study
TSW27 (mNeonGreen single copy)	*mbbSi1 [ rgef-1 p::mNeongreen:: let-858 -3’UTR + Cbr- unc-119 ] II *	This study
TSW47 (mEYFP overexpression)	*mbbIs12 [ rgef-1 p::FLAG::mEYFP:: let-858 -3’UTR] *	This study
TSW58 (mEYFP single copy)	*mbbSi6 [ rgef-1 p::FLAG::mEYFP:: let-858 -3’UTR + Cbr- unc-119 ] II *	This study
AM52	*rmIs182 [ rgef-1 p::Q0::YFP] *	Morimoto lab

## Extended Data

Description: Extended Data Figure S1. Resource Type: Image. DOI: 10.22002/wr39k-0n228
